# The complete chloroplast genome sequence of *Gossypium hirsutum*: organization and phylogenetic relationships to other angiosperms

**DOI:** 10.1186/1471-2164-7-61

**Published:** 2006-03-23

**Authors:** Seung-Bum Lee, Charalambos Kaittanis, Robert K Jansen, Jessica B Hostetler, Luke J Tallon, Christopher D Town, Henry Daniell

**Affiliations:** 1 Dept. of Molecular Biology & Microbiology, University of Central Florida, Biomolecular Science, Building #20, Orlando, FL 32816–2364, USA; 2 Section of Integrative Biology and Institute of Cellular and Molecular Biology, Patterson Laboratories 141, University of Texas, Austin, TX 78712, USA; 3 The Institute for Genomic Research, 9712 Medical Center Drive, Rockville, MD 20850, USA

## Abstract

**Background:**

Cotton (*Gossypium hirsutum*) is the most important fiber crop grown in 90 countries. In 2004–2005, US farmers planted 79% of the 5.7-million hectares of nuclear transgenic cotton. Unfortunately, genetically modified cotton has the potential to hybridize with other cultivated and wild relatives, resulting in geographical restrictions to cultivation. However, chloroplast genetic engineering offers the possibility of containment because of maternal inheritance of transgenes. The complete chloroplast genome of cotton provides essential information required for genetic engineering. In addition, the sequence data were used to assess phylogenetic relationships among the major clades of rosids using cotton and 25 other completely sequenced angiosperm chloroplast genomes.

**Results:**

The complete cotton chloroplast genome is 160,301 bp in length, with 112 unique genes and 19 duplicated genes within the IR, containing a total of 131 genes. There are four ribosomal RNAs, 30 distinct tRNA genes and 17 intron-containing genes. The gene order in cotton is identical to that of tobacco but lacks *rpl22 *and *infA*. There are 30 direct and 24 inverted repeats 30 bp or longer with a sequence identity ≥ 90%. Most of the direct repeats are within intergenic spacer regions, introns and a 72 bp-long direct repeat is within the *psaA *and *psaB *genes. Comparison of protein coding sequences with expressed sequence tags (ESTs) revealed nucleotide substitutions resulting in amino acid changes in *ndhC, rpl23, rpl20, rps3 *and *clpP*. Phylogenetic analysis of a data set including 61 protein-coding genes using both maximum likelihood and maximum parsimony were performed for 28 taxa, including cotton and five other angiosperm chloroplast genomes that were not included in any previous phylogenies.

**Conclusion:**

Cotton chloroplast genome lacks *rpl22 *and *infA *and contains a number of dispersed direct and inverted repeats. RNA editing resulted in amino acid changes with significant impact on their hydropathy. Phylogenetic analysis provides strong support for the position of cotton in the Malvales in the eurosids II clade sister to *Arabidopsis *in the Brassicales. Furthermore, there is strong support for the placement of the Myrtales sister to the eurosid I clade, although expanded taxon sampling is needed to further test this relationship.

## Background

The chloroplast is the site of photosynthesis, where light energy in photons is converted into chemical bond energy, via redox reactions, including inorganic carbon fixation at Calvin's cycle, finally yielding energy-rich carbohydrate molecules. Therefore, apart from the antennae, photosystem I and II complexes, which are found in the thylakoid membrane, the chloroplast contains the entire enzymatic machinery for carbohydrate biosynthesis in the stroma. Anabolic pathways such as protein, fatty acid, vitamin, and pigment biosynthesis take place in the chloroplast as well, indicating the organelle's ability to synthesize complex molecules. The chloroplast genome maintains a highly conserved organization [1,2] with most land plant genomes composed of a single circular chromosome with a quadripartite structure that includes two copies of an inverted repeat (IR) that separate the large and small single copy regions (LSC and SSC) [3]. The recent surge of interest in sequencing chloroplast genomes has provided a plethora of information on the organization and evolution of these genomes and new data for reconstructing phylogenetic relationships [2].

Chloroplast genetic engineering offers numerous advantages, including a high-level of transgene expression [4], multi-gene engineering in a single transformation event [4-7], transgene containment via maternal inheritance [8-11] or cytoplasmic male sterility [12], lack of gene silencing [4,13], position effect due to site specific transgene integration [14], and pleiotropic effects due to sub-cellular compartmentalization of transgene products [13,15,16]. Apart from expressing therapeutic agents, biopolymers, or transgenes to confer agronomic traits, plastid genetic engineering has been used to study plastid biogenesis and function, revealing mechanisms of plastid DNA replication origins, intron maturases, translation elements and proteolysis, import of proteins and several other processes [18]. Despite the potential of chloroplast genetic engineering, this technology has only recently been extended to the major crops, including soybean [19], carrot [20] and cotton [21], via somatic embryogenesis, achieving transgene expression in non-green plastids [22]. All other previous studies focused on direct organogenesis by bombardment of leaves containing mature green chloroplasts [22]. Lack of complete chloroplast genome sequences to provide 100% homologous species-specific chloroplast transformation vectors, containing suitable selectable markers and endogenous regulatory elements, is one of the major limitations to extend this concept to other useful crops [22,23].

The need for sequencing the cotton plastome is obvious, when considering its annual retail value of about $120 billion, making it America's most value-added crop. This is justified by the fact that cotton is the single most important textile fiber grown in 90 countries; the US accounts for 21% of the total world fiber production. In 2004–2005, US farmers planted 79% of the 5.7-million hectares of nuclear transgenic cotton. Upland cotton, *Gossypium hirsutum*, has the potential to hybridize with *G. tomentosum*, feral populations of *G. hirsutum*, and *G. hirsutum/G. barbadense *[21]. Therefore, geographical restrictions in planting genetically modified cotton are in place because of reports of pollen dispersal from transgenic cotton plants [25]. Chloroplast genetic engineering could minimize transgene escape because of maternal inheritance of transgenes [8-11]. In addition, other failsafe mechanisms, including cytoplasmic male sterility could be employed to contain transgenes [12].

The examination of phylogenetic relationships among angiosperms has received considerable attention during the past decade [reviewed in [26]]. Although there is considerable consensus about the circumscription and relationships among many of the major clades, most molecular phylogenetic analyses have examined numerous taxa but have relied on only a few gene sequences. Completely sequenced chloroplast genomes provide a rich source of nucleotide sequence data that can be used to address phylogenetic questions. Several recent studies have attempted to use completely sequenced genomes to resolve the identification of the basal lineages of flowering plants [27-29]. Use of many or all of the genes from the chloroplast genome provides many more characters for phylogeny reconstruction in comparison with previous studies that have relied on only a few genes. However, the limited number of available whole chloroplast genome sequences can result in misleading estimates of relationship [27,30]. This problem can be overcome as more complete chloroplast genome sequences become available.

In this article, we present the complete sequence of the chloroplast genome of upland cotton, *Gossypium hirsutum*. One goal of this paper is to examine gene content and gene order, and determine the distribution and location of repeated sequences. Secondly, the RNA editing sites in the cotton chloroplast genome are identified and examined, by comparing the DNA sequences with available expressed sequence tag (EST) sequences, because RNA editing plays a major role in several lineages of plants [31,32]. Lastly, protein-coding sequences from 61 genes are used to estimate phylogenetic relationships of cotton with 25 other angiosperms.

## Results

### Size, gene content, order and organization of the cotton chloroplast genome

**Figure 1:**
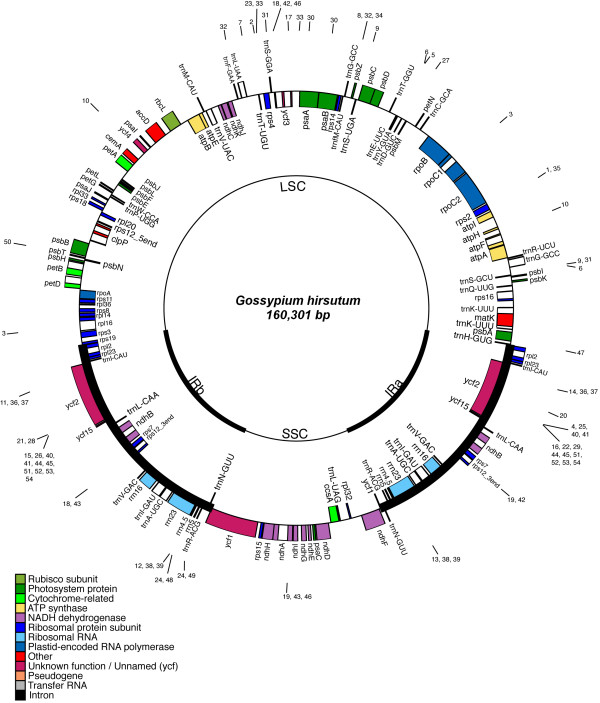
Gene map of the *Gossypium hirsutum *chloroplast genome. The thick lines indicate the extent of the inverted repeats (IRa and IRb), which separate the genome into small (SSC) and large (LSC) single copy regions. Genes on the outside of the map are transcribed in the clockwise direction and genes on the inside of the map are transcribed in the counterclockwise direction. Numbered lines around the map indicate the location of repeated sequences found in the cotton genome (see Table 1 for details). The SSC region is in the reverse orientation relative to tobacco [80]. This does not reflect any differences in gene order for cotton but simply reflects the well-known phenomenon that the SSC exists in two orientations in chloroplast genomes [84].

Cotton complete chloroplast genome is 160,301 bp in length (Fig. 1), and includes a pair of inverted repeats 25,608 bp long, separated by a small and a large single copy region of 20,269 bp and 88,816 bp, respectively. There are 112 unique genes within the cotton chloroplast genome and 19 of these are duplicated in the IR, giving a total of 131 genes (Fig. 1). Furthermore, there are four ribosomal and 30 distinct tRNA genes; seven of the tRNA genes and all rRNA genes are duplicated within the IR. There are 17 intron-containing genes, 15 of which contain one intron, whereas the remaining two have two introns. The gene order in the cotton plastid genome is identical to that of tobacco, but cotton lacks the *rpl22 *and *infA *genes. Overall, genomic content is 37.25% GC and 62.75% AT, where 56.46% of the genome corresponds to protein coding genes and 43.54% to non-coding regions, including introns and intergenic spacers.

### Repeat structure

**Figure 2:**
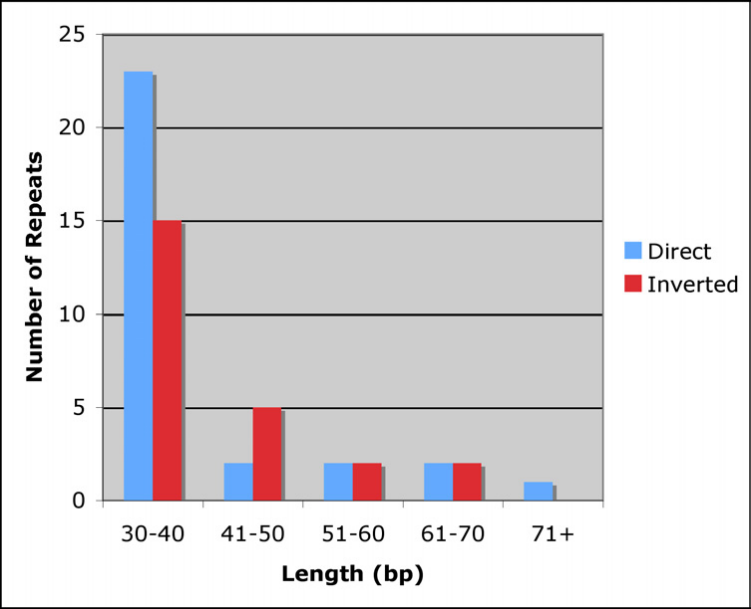
Histogram showing the number of repeated sequences ≥ 30 bp long with a sequence identity ≥ 90% in the cotton chloroplast genome.

Repeat analysis identified 30 direct and 24 inverted repeats 30 bp or longer with a sequence identity of at least 90% (Fig. 2 and Table 1). Twenty-three direct and 15 inverted repeats are 30 to 40 bp long, and the longest direct repeat is 72 bp. Most of the direct repeats are within intergenic spacer regions, intron sequences and *ycf2*, an essential hypothetical chloroplast gene [33]. Interestingly, a 72 bp-long direct repeat was found in the *psaA *and *psaB *genes, whereas a 34-bp forward repeat was within the *rrn23 *gene, and a shorter, 32 bp-long direct repeat was identified in two serine transfer-RNA(*trnS*) genes that recognize different codons; *trnS-GCU *and *trnS-UGA*.

### RNA editing

Comparison of the nucleotide sequences of protein coding genes and EST sequences retrieved from GenBank revealed that *rps16, rpl2, rpoC2, rps4 *and *ycf1 *have 100% sequence identity with their respective ESTs (data not shown). Eleven non-synonymous nucleotide substitutions, resulting in a total of nine amino acid changes, were identified within *ndhC, rpl23, rpl20, rps3 *and *clpP *compared to respective ESTs, although their sequence identity was above 98% (Table 2). Surprisingly, there were no synonymous substitutions. All of the five aforementioned genes experienced one or two nucleotide substitutions, apart from the protease-encoding *clpP*, which had five variable sites. Lastly, in all but *rpl23*, the nucleotide substitutions had an impact on the hydropathy of the amino acid because they changed the amino acids from aliphatic to hydrophilic, and vice versa.

### Phylogenetic analysis

**Figure 3:**
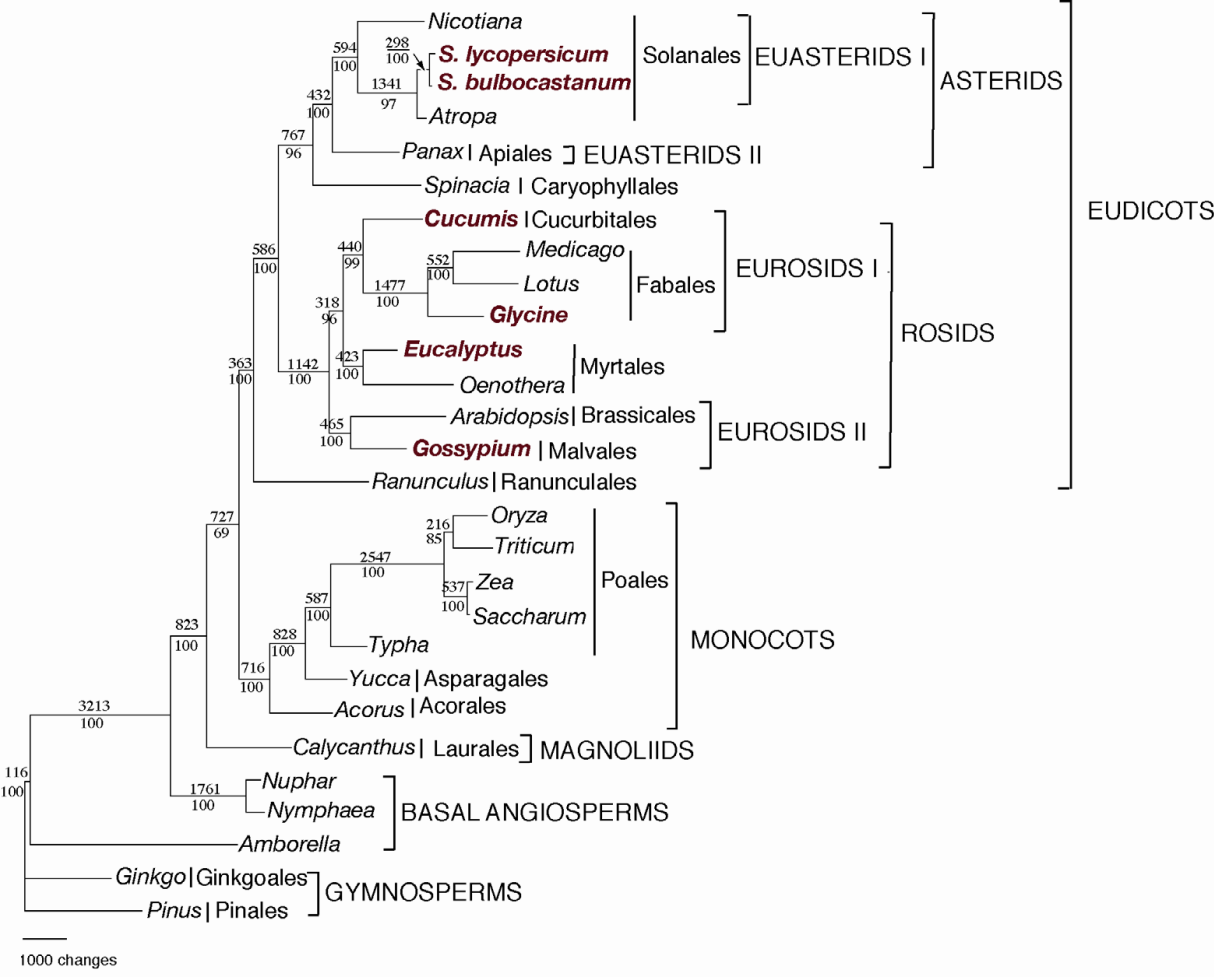
Parsimony tree based on 61 chloroplast protein-coding genes. The tree has a length of 49,957, a consistency index of 0.46 (excluding uninformative characters) and a retention index of 0.6. Numbers above node indicate number of changes along each branch and numbers below nodes are bootstrap support values. Taxa in red are those which have not appeared in any previous phylogenetic studies using 61 genes from complete chloroplast genome sequences. Ordinal and higher level group names follow APG II [85]. The maximum likelihood tree has the same topology but is not shown.

The data matrix for phylogenetic analyses included 61 protein-coding genes for 28 taxa (Table 3), including 26 angiosperms and two gymnosperm outgroups (*Pinus *and *Ginkgo*). The data set comprised 45,573 nucleotide positions but when the gaps were excluded there were 39,624 characters. Maximum Parsimony (MP) analyses resulted in a single, fully resolved tree with a length of 49,957, a consistency index of 0.46 (excluding uninformative characters) and a retention index of 0.62 (Fig. 3). Bootstrap analyses indicated that 24 of the 26 nodes were supported by values ≥ 95% with 19 of these with bootstrap values of 100%. Maximum Likelihood (ML) analysis resulted in a tree with a –lnL = 311251.33. The ML and MP trees had identical topologies so only the MP tree is shown in Figure 3.

Several major groups were supported within angiosperms and these groups are generally in agreement with recent classifications [26]. The most basal lineage was *Amborella *followed by the Nymphaeales. The next branch included *Calycanthus*, the sole representative of magnoliids in the data set. This was followed by a strongly supported clade of monocots, represented by members of three different orders (Acorales, Asparagales, and Poales). The monocots were then sister to the eudicots with the Ranunculales forming the earliest diverging eudicot clade. Within the core eudicots there were two major clades, one including the rosids and the second including the Caryophyllales sister to asterids. Within the rosid clade there were two major groups, the eurosids II and a group that included the Myrtales sister to the eurosids I. *Gossypium *in the Malvales was sister to *Arabidopsis *in the Brassicales.

## Discussion

### Implications for integration of transgenes

We have recently demonstrated stable transformation of the cotton plastid genome and maternal inheritance of transgenes via somatic embryogenesis [21]. In contrast to previous reports on integrating foreign genes in tomato and potato chloroplast genomes using tobacco flanking sequences that do not have 100% sequence identity [24,34,35], the cotton plastid transformation vector was constructed using the PCR-amplified native cotton 16S/*trnI*-*trnA/*23S sequence. However, regulatory sequences used in the cotton plastid transformation were derived from tobacco or other heterologous sequences. With the availability of the entire cotton chloroplast genome sequence, it should now be possible to utilize endogenous regulatory sequences. Species-specific vectors should be effective for plastid transformation, especially in recalcitrant plants, because of transgene integration using flanking sequences with 100% sequence identity and endogenous promoters, 5' & 3'untranslated regions, thereby enhancing transcription and translation of transgenes. Also, the complete chloroplast genome provides the option of transgene integration into transcriptionally silent, active or read-through spacer regions for optimal transgene integration.

Thus far, transgenes conferring several useful agronomic traits, including insect [4,36,37], herbicide [8,38], and disease resistance [39], drought [13] and salt tolerance [20], phytoremediation [5], as well as cytoplasmic male sterility [12], have been stably integrated and expressed, via the tobacco chloroplast genome. Using the chloroplast as a bioreactor, vaccine antigens [15,40-42], human therapeutic proteins [17,43-45], industrial enzymes [46] and biomaterials [6,47,48] have been produced successfully in an environmental friendly way. Although many successful examples of plastid engineering in tobacco have set a solid foundation for various future applications, this technology has not been extended to many of the major crops, primarily due to the lack of complete chloroplast genome sequences and challenges in achieving homoplasmy in recalcitrant crops.

### Evolutionary implications

Other than the IR, repeated sequences are generally considered to be uncommon in chloroplast genomes [1]. Furthermore, previous studies based on both filter hybridization and DNA sequencing have indicated that dispersed repeats are found more commonly in genomes that have experienced changes in genome organization [49,56], especially in highly rearranged algal genomes [51,52]. The most extensive examination of repeat structure in angiosperms was performed in legumes [3], which do have a single inversion and in some taxa a loss of one copy of the IR. These repeat analyses identified a substantial number highly conserved repeats ≥ 30 bp with a sequence identity of ≥ 90%. Many of these repeats were located in intergenic spacer regions and introns, with several located in the coding regions of *psaA*, *psaB*, and *ycf2*. Our examination of repeats in the cotton chloroplast genome (Table 1, Fig. 2) identified similar numbers of repeats as in legumes [3], and these are also located mostly in intergenic spacer regions and introns. Repeats in coding regions of cotton are located in the same genes as in legumes. Overall, it appears that dispersed repeats are very common in angiosperm chloroplast genomes, even in genomes that have not experienced rearrangements. Future comparative studies are needed to determine the functional and evolutionary role these repeats may play in chloroplast genomes.

DNA and EST sequence comparisons identified many nucleotide substitutions resulting in amino acid changes. Based on previous studies of *Atropa *[53] and tobacco [54], posttranscriptional RNA editing events result predominantly in C-to-U edits. However, analysis of the cotton genome and EST sequences indicates that only two of the eleven differences were C-to-U changes, suggesting that most of these changes are not mRNA edits but may simply represent intra-species polymorphisms. Evolutionary loss of RNA editing sites has been previously observed and could possibly be due to a decrease in the effect of RNA-editing enzymes [31]. Additionally, conversions other than C-to-U in cotton, as well as other crops, suggest that chloroplast genomes may be accumulating considerable amounts of nucleotide substitutions, where some genes might accrue more alterations than others, such as the *petL *and *ndh *genes that have a high frequency of RNA editing [55]. Therefore, despite the plastome's high conservation, variations occur post-transcriptionally, promoting translational efficiency due to transcript-protein complex binding and/or changes in the chloroplast microenvironment, like redox potential or light intensity [56,57].

The phylogeny based on 61 protein-coding genes for 28 angiosperms is congruent with relationships suggested in previous studies [summarized in [26]]. There is strong support for the monophyly all of the major clades of angiosperms, including monocots, eudicots, rosids, asterids, eurosids I, eurosids II, asterids I and asterid II. Our phylogenetic analyses have greatly expanded the taxon sampling of entire genomes because we included six genomes (in bold in Table 1 and Fig. 3) that have not been included in recently published phylogenies based on complete chloroplast genomes [27-29,58]. The sampling is particularly expanded in the rosids with four of the six genomes from this clade. Thus, we will focus our discussion of the phylogenetic implications of this expanded analysis on this group.

The rosid clade is very large and includes nearly 140 families representing almost one third of all angiosperms. The most recent phylogenies of this group [summarized in chapter 8 in [26]] indicate that there are seven major clades whose relationships still remain unresolved. Representatives of three of these major clades are included in our analyses, eurosids I, eurosids II, and Myrtales. The position of the Myrtales has been especially controversial with no clear resolution of the relationship of this order to other members of the rosids. Our 61 gene chloroplast phylogeny (Fig. 3) provides strong support for a sister relationship of the Myrtales with the eurosid I clade. A three-gene phylogeny of 560 angiosperms is congruent with our results [59], although support was very weak. However, a sister relationship between eurosids I and Myrtales is in conflict with two other recent phylogenies based on two chloroplast genes (*atpB*, *rbcL*), which placed the Myrtales sister to the eurosid II clade with weak support [60,61]. Although our results clearly favor a closer relationship of Myrtales to the eurosid I clade, expanded sampling of complete chloroplast genome sequences of rosids is needed to resolve this issue, especially since limited taxon sampling can lead to erroneous tree topologies [27,30].

Our chloroplast phylogeny (Fig. 3) also supports the sister relationship between the orders Cucurbitales and Fabales, two of the four nitrogen fixing clades of eurosids I. Furthermore, the position of cotton, a member of the order Malvales, as sister to *Arabidopsis *in the Brassicales, is in agreement with recently phylogenies of the eurosid II clade [26].

## Conclusion

Our complete sequence of the cotton chloroplast genome provides the needed information for expanding chloroplast genetic engineering to this important crop plant. Although genome organization of cotton is very similar to other unrearranged angiosperm chloroplast genomes, identification of disperse repeats and potential RNA editing sites provides new insights into the evolution of this genome. Finally, phylogenetic analyses of sequences of 61 protein-coding genes for 26 angiosperms suggests that the order Myrtales is sister to the eurosid I clade but denser sampling is needed to test this result rigorously.

## Methods

### DNA isolation and amplification

*Gossypium hirsutum *plants cv. Coker310FR were grown from seedlings in soil pots, until they were 1 m tall. Prior to DNA extraction, the plants were placed in the dark for two days to reduce the chloroplast starch levels. After that, 10 g of young leaf tissue was collected for cpDNA isolation based on the sucrose step gradient centrifugation method by Sandbrink *et al *[62]. Isolation was followed by whole chloroplast genome Rolling Circle Amplification (RCA), using the Repli-g RCA kit (Qiagen, Inc.) following the methods outlined in [63]. After incubation at 30°C for 16 hr, the reaction was terminated with 10-minute incubation at 65°C. Digestion of the RCA product with *BstXI, EcoRI *and *HindIII *allowed verification of successful RCA plastome amplification, as well as assessment of its quality, prior to DNA sequencing.

### DNA sequencing and genome assembly

DNA was sheared by nebulization, size fractionated to 4–6 kb, linker ligated and cloned into pHOS2, a TIGR medium copy vector. A total of 1619 good reads with an average length of 812 bases was generated during the random (1396 reads) and closure (223 reads) phases of sequencing. Sequences were assembled using TIGR assembler [64] and scaffolded using Bambus [65]. Sequence finishing included directed PCR to span gaps, directed primer walking on clones and transposon mediated sequencing of full clones to cover the entire genome and complete regions of low coverage and manual editing of sequences to resolve inconsistencies.

### Gene annotation

The cotton genome was annotated using DOGMA [Dual Organellar GenoMe Annotator, [66]], after uploading a FASTA-formatted file of the complete plastid genome to the program's server. BLASTX and BLASTN searches, against a custom database of previously published plastid genomes, identified cotton's putative protein-coding genes, and tRNAs or rRNAs. For genes with low sequence identity, manual annotation was performed, after identifying the position of the start and stop codons, as well as the translated amino acid sequence, using the plastid/bacterial genetic code.

### Examination of repeat structure

REPuter [67] was used to locate and count the direct (forward) and inverted (palindromic) repeats within the cotton chloroplast genome. For repeat identification, the following constraints were used: (i) minimum repeat size of 30 bp, and (ii) 90% or greater sequence identity, based on Hamming distance equal to 3 bp [3]. Manual verification of the identified repeats was performed in EditSeq, while performing intragenomic blast search of the identified repeat sequence.

### Variation between coding sequences and cDNAs

Each of the gene sequences from the cotton chloroplast genome was used to perform a BLAST search of expressed sequence tags (ESTs) from GenBank. The retrieved *Gossypium hirsutum *ESTs were aligned with the corresponding annotated gene using ClustalX [68], followed by screening for nucleotide and amino acid changes using Megalign and its' plastid/bacterial genetic code. Because of variation in the length between an EST and the related gene, the length of the analyzed sequence was recorded.

### Phylogenetic analysis

The 61 genes included in the analyses of Goremykin et al. [28,29] and Leebens-Mack et al. [27] were extracted from our new chloroplast genome sequences of cotton using the organellar genome annotation program DOGMA. [66]. The same set of 61 genes was extracted from chloroplast genome sequences of five other recently sequenced angiosperm chloroplast genomes, including tomato, potato, soybean, cucumber, and *Eucalyptus *(see Table 3 for complete list of genomes examined). In general, alignment of the DNA sequences was straightforward and simply involved removing gaps included in the data set because of the elimination of non-seed plants and adding the 61 genes for the new angiosperms to the aligned data matrix from Leebens-Mack et al. [27]. In some cases, small in frame insertions or deletions were required for correct alignment. For two genes, *ccsA *and *matK*, the DNA sequences were more divergent, requiring alignment using ClustalX [68] followed by manual adjustments.

Phylogenetic analyses using maximum parsimony (MP) and maximum likelihood (ML) were performed using PAUP* version 4.10 [69]. All phylogenetic analyses excluded gap regions. All MP searches were heuristic with 100 random addition replicates and TBR branch swapping with the Multrees option. The Hasegawa-Kishino-Yano (HKY; [70]) model of molecular evolution was used in ML analyses of the nucleotide sequences. ML analyses used TBR branch swapping with the Multrees option and one random addition replicate. Non-parametric bootstrap analyses [71] were performed for MP analyses with 1000 replicates with TBR branch swapping, one random addition replicate, and the Multrees option and for ML analyses with 100 replicates with NNI branch swapping, one random addition replicate, and the Multrees option.

## Abbreviations

cpDNA, chloroplast DNA; IR inverted repeat; SSC, small single copy; LSC, large single copy, bp, base pair; ycf, hypothetical chloroplast reading frame; rrn, ribosomal RNA; MP, maximum parsimony; ML, maximum likelihood; EST, expressed sequence tag; cDNA, complementary DNA.

## Authors' contributions

SBL isolated chloroplasts, performed RCA amplification of cpDNA, genome annotation, analysis and submission of data to the GenBank; CK performed the repeat analyses, comparisons of DNA and EST sequences, assisted with extraction & alignment of DNA sequences for phylogenetic analyses and wrote a few sections of the first draft; JBH, LJT and CDT performed DNA sequencing and genome assembly; RKJ assisted with extracting and aligning DNA sequences, performed phylogenetic analyses, and wrote the phylogenetic portions of the manuscript; HD conceived and designed this study, interpreted data, wrote and revised several versions of this manuscript. All authors read and approved the final manuscript.

## Figures and Tables

**Table 1:** Location of identified repeats in the cotton plastid genome. Table includes repeats at least 30 bp in size, with a sequence identity greater than or equal to 90%. IGS = Intergenic spacer. See Fig. 1 for location of repeats on the gene map.

**Repeat Number**	**Size (bp)**	**Location**
1	30	IGS
2	30	IGS
3	30	*rpoC1 *intron, *rpl16 *intron
4	30	*ycf2*
5	31	IGS
6	32	*psbI *(5 bp) – IGS, IGS
7	32	IGS
8	32	IGS
9	32	IGS (4 bp) – *trnS-GCU*, IGS (4 bp) – *trnS-UGA*
10	32	IGS
11	34	*ycf2*
12	34	IGS
13	34	*rrn23 *exon
14	34	*ycf2*
15	34	*ycf2*
16	34	*ycf2*
17	35	*ycf3 *intron
18	36	*ycf3 *intron, IGS
19	38	*ndhA *intron, *rps12_3end *intron
20	38	*ycf2*
21	38	*ycf2*
22	38	*ycf2*
23	40	IGS
24	43	IGS
25	47	*ycf2*
26	52	*ycf2*
27	58	IGS
28	64	*ycf2*
29	64	*ycf2*
30	72	*psaA *exon, *psaB *exon
31	30	IGS (2 bp) – *trnS-GCU*, *trnS-GGA*
32	30	IGS
33	30	IGS
34	31	IGS
35	34	IGS
36	34	*ycf2*
37	34	*ycf2*
38	34	IGS
39	34	IGS
40	34	*ycf2*
41	34	*ycf2*
42	36	*ycf3 *intron, IGS
43	38	IGS, *ndhA *intron
44	38	*ycf2*
45	38	*ycf2*
46	41	*ycf3 *intron, *ndhA *intron
47	41	IGS
48	43	IGS
49	43	IGS
50	48	IGS
51	52	*ycf2*
52	52	*ycf2*
53	64	*ycf2*
54	64	*ycf2*

**Table 2:** Differences observed by comparison of cotton chloroplast genome sequences with EST sequences obtained by BLAST searches of GenBank.

**Gene**	**Gene size (bp)**	**Sequence analyzed^a^**	**Number of variable sites**	**Variation type**	**Position(s)^b^**	**Amino acid change**
*clpP*	591	228–537	5	A-G	523	M-A
				T-C	524	
				T-A	528	I-M
				T-G	531	G-S
				G-A	532	
*ndhC*	363	76–363	1	T-C	323	L-S
*rpl20*	354	1–354	2	A-G	263	K-R
				C-U	308	S-L
*rpl23*	282	85–282	1	C-U	89	S-L
*rps3*	657	274–657	2	T-G	275	L-R
				A-C	302	K-T

^**a**^Sequence analyzed coordinates based on the gene sequence, considering the first base of the initiation codon as bp 1. ^**b**^Variable position is given in reference to the first base of the initiation codon of the gene sequence.

**Table 3:** Taxa included phylogenetic analyses with GenBank accession numbers and references. Taxa in bold are those which have not appeared in any previous phylogenetic studies using 61 genes from complete chloroplast genome sequences.

Taxon	GenBank Accession Numbers	Reference
Gymnosperms –Outgroups		
*Pinus thunbergii*	NC_001631	Wakasugi et al. 1994 [72]
*Ginkgo biloba*	DQ069337–DQ069702	Leebens-Mack et al 2005 [27]
Basal Angiosperms		
*Amborella trichopoda*	NC_005086	Goremykin et al. 2003 [29]
*Nuphar advena*	DQ069337–DQ069702	Leebens-Mack et al 2005 [27]
*Nymphaea alba*	NC_006050	Goremykin et al. 2004 [28]
Monocots		
*Acorus americanus*	DQ069337–DQ069702	Leebens-Mack et al 2005 [27]
*Oryza sativa*	NC_001320	Hiratsuka et al. 1989 [73]
*Saccharum officinarum*	NC_006084	Asano et al. 2004 [74]
*Triticum aestivum*	NC_002762	Ikeo and Ogihara, unpublished
*Typha latifolia*	DQ069337–DQ069702	Leebens-Mack et al 2005 [27]
*Yucca schidigera*	DQ069337–DQ069702	Leebens-Mack et al 2005 [27]
*Zea mays*	NC_001666	Maier et al. 1995 [75]
Magnoliids		
*Calycanthus floridus*	NC_004993	Goremykin et al. 2003 [76]
Eudicots		
*Arabidopsis thalliana*	NC_000932	Sato et al. 1999 [77]
*Atropa belladonna*	NC_004561	Schmitz-Linneweber et al. 2002 [53]
***Cucumis sativus***	NC_007144	**Plader et al. unpublished**
***Eucalyptus globulus***	AY780259	**Steane 2005 [78]**
***Glycine max***	DQ317523	**Saski et al. 2005 [3]**
***Gossypium hirsutum***	DQ345959	**Current study**
*Lotus corniculatus*	NC_002694	Kato et al. 2000 [79]
*Medicago truncatula*	NC_003119	Lin et al., unpublished
*Nicotiana tabacum*	NC_001879	Shinozaki et al. 1986 [80]
*Oenothera elata*	NC_002693	Hupfer et al. 2000 [81]
*Panax schinseng*	NC_006290	Kim and Lee 2004 [82]
*Ranunculus macranthus*	DQ069337–DQ069702	Leebens-Mack et al 2005 [27]
***Solanum lycopersicum***	DQ347959	**Daniell et al. in press**
***Solanum bulboscastanum***	DQ347958	**Daniell et al. in press**
*Spinacia oleracea*	NC_002202	Schmitz-Linneweber et al. 2001 [83]
